# Expression of Dickkopf-1 and Beta-Catenin Related to the Prognosis of Breast Cancer Patients with Triple Negative Phenotype

**DOI:** 10.1371/journal.pone.0037624

**Published:** 2012-05-23

**Authors:** Wen-Huan Xu, Zhe-Bin Liu, Chen Yang, Wenxin Qin, Zhi-Ming Shao

**Affiliations:** 1 Department of Breast Surgery, Breast Cancer Institute, Cancer Hospital, Fudan University, Shanghai, People’s Republic of China; 2 Department of Oncology, Shanghai Medical College, Fudan University, Shanghai, People’s Republic of China; 3 Institutes of Biomedical Science, Fudan University, Shanghai, People’s Republic of China; 4 National Laboratory for Oncogenes and Related Genes, WHO Collaborating Center for Research on Cancer, Shanghai Cancer Institute, Shanghai Jiao Tong University School of Medicine, Shanghai, People’s Republic of China; University of Navarra, Spain

## Abstract

**Background and Aim:**

We investigated the prognostic importance of dickkopf-1(DKK1) and beta-catenin expression in triple negative breast cancers.

**Methods:**

The expression of DKK1 and beta-catenin was evaluated in breast cell lines using RT-PCR and western blot. Immunohistochemistry was used to characterize the expression pattern of DKK1 and beta-catenin in 85 triple negative breast cancers and prognostic significance was assessed by Kaplan-Meier analysis and Cox proportional hazards regression modeling.

**Results:**

The expression of DKK1 was confirmed in hormone-resistant breast cell lines MDA-MB-231, MDA-MB-231-HM and MDA-MB-435. Expression of DKK1 in triple negative breast cancers correlated with cytoplasmic/nuclear beta-catenin (p = 0.000). Elevated expression of DKK1 and cytoplasmic/nuclear beta-catenin in triple negative cancers indicate poor outcome of patients. DKK1 was also a prognostic factor for patients with earlier stage or no lymph node metastasis.

**Conclusion:**

DKK1 together with beta-catenin might be important prognostic factors in triple negative breast carcinoma. DKK1 might be a valuable biomarker in predicting the prognosis of patients with earlier stage or no lymph node metastasis. It is possible that through further understanding of the role of Wnt/beta-catenin pathway activation, beta-catenin would be a potential therapeutic target for the triple negative breast cancer.

## Introduction

Breast cancer is a disease with heterogeneous nature and our understanding of the breast subtypes had been previously based on histopathology and immunohistochemical criteria. Over the last decade, gene expressing studies with DNA microarrays have identified four common subtypes of this disease. ER positive tumors included luminal A and B tumors, while ER negative tumors included basal-like and HER-2 positive tumors. Most of the basal-like tumors, which also referred to as “triple-negative” breast cancers, are negative for ER, PR and HER2[1], [2].

Women with triple-negative breast cancers are reported to have an increased likelihood of distant recurrence and death than women with other types of breast cancer within 5 years after diagnosis but not there-after [3]. They also have been reported to have a tendency toward visceral (versus bone) metastasis, local relapse and cerebral metastases [4], [5]. Since the usage of trastuzumab and lapatinib, the recurrence and mortality rate of HER2 positive patients has considerably reduced, which may render the triple negative women at most risk of early recurrence. The prognosis of breast cancer patients may be influenced by traditional parameters, such as pathology type, lymph node status, tumor size, stage and expression of hormone receptors. Yet patients with the same parameters mentioned above may have totally different prognosis. So biomarkers for the prediction of prognosis are need in daily clinical practice.

The Wnt/beta-catenin pathway was implicated in mammary tumorigenesis since over expression of wnt1 gene in the mammary epithelium was identified sufficient for mammary gland hyperplasia and adeno-carcinomas [6]. Binding to the frizzled receptor (Fz) and the low-density lipoprotein receptor-related protein-5/6(LRP5/6), Wnt-1 protein prevents phosphorylation and degradation of beta-catenin by the GSK3β/APC/axin destruction complex. Subsequently accumulated cytosolic and nuclear beta-catenin bound to TCF transcription factors and resulted in activating downstream signals which are important for proliferation and matrix remodeling [7]. Studies in mice strongly suggest that deregulated beta-catenin signaling increases risk of breast cancer by inducing stem cell and early progenitor cell accumulation [8], [9]. And one gene expression signature derived from MMTV-Wnt1 tumor-initiating cells was found to have prognostic value in basal-like and hormone receptor-negative cancers [10]. Several studies have reported increased cytoplasmic and nuclear beta-catenin in primary breast cancers, especially basal-like breast cancers, and correlated with poor prognosis and survival [11], [12], [13].

**Figure 1:**
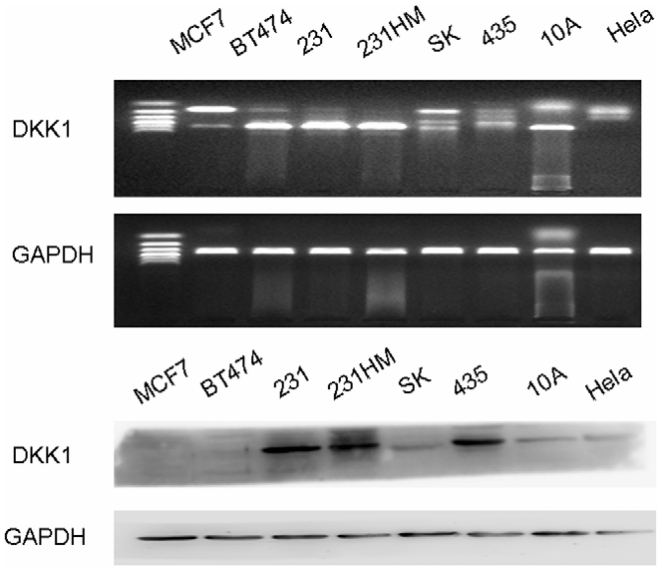
The mRNA and protein expression of DKK1 in breast cancer cell lines. A) The mRNA expression of DKK-1 in MCF-7, BT474, MDA-MB231, MDA-MB231HM, SK, MDA-MB-435, MCF-10A and Hela cells. B) The expression of DKK1 in MCF-7, BT474, MDA-MB231, MDA-MB231HM, SK, MDA-MB-435, MCF-10A and Hela cells by western blot.

**Table 1:** Clinical and histopathological features in 85 triple negative patients.

Feature	No.	Rate(%)
Pathology type		
IDC	63	74.12%
Other	22	25.88%
Lymph node status		
0	54	63.53%
> = 1	31	36.47%
Tumor size		
< = 2 cm	30	35.29%
>2 cm	55	64.71%
Tumor stage		
Stage1,2	62	72.94%
Stage3	23	27.06%
Menopausal status		
Pre	41	48.24%
Post	44	51.76%
TOTAL	85	100%

IDC, invasive ductal carcinoma;

DKK-1, a secreted protein involved in head formation in embryonic development, binds to LRP5/6, blocks Wnt-1 protien signaling and plays as an antagonist of Wnt signaling [14]. DKK1 has also been reported to be over expressed in many carcinomas including lung cancer, esophageal squamous cell carcinoma, hepatocellular carcinoma, Wilm’s tumor and multiple myeloma [15], [16], [17], [18]. These indicate that DKK1 performs as an oncogenic factor rather than a tumor-suppressor in tumors. Forget MA et al has reported expression of DKK1 in 21 out of 73 cases of breast cancer patients, in particular hormone-resistant patients [19]. These findings raise an intriguing possibility that DKK1 may be involved in the prognosis in triple negative breast cancer. To test this hypothesis, we investigated the expression of DKK1 and beta-catenin in tumor specimens from 85 patients with breast cancer and the correlation between DKK1 and beta-catenin together with other clinical pathological features of prognosis in this study.

## Materials and Methods

### Ethics

All samples were anonymously coded in accordance with local ethical guidelines, and written informed consent was obtained. This study was approved by the Review Board of Fudan University Shanghai Cancer Center and conforms to the principles outlined in the Declaration of Helsinki (IRB number: 050432-4-10087A).

### Cell Lines

Human breast cancer cell lines MDA-MB-231, MCF7, BT-474, SK-BR-3, MDA-MB-435, MCF10A and Hela cell were purchased from the American Type Culture Collection. The highly metastatic MDA-MB-231HM line, which has a high potential to metastasize to the lung, was established by our institute [20].

### Patients and Specimens

A total of 1614 different breast invasive carcinoma was identified in the database of the Department of Breast Surgery of the Cancer Hospital affiliated to Fudan University in the period between 2000 and 2002. We targeted the 136 triple-negative (ER negative, PR (progesterone receptor)-negative, HER-2 negative) patients who were consecutively obtained from the 1614 cases with operable breast cancer. They have been followed regularly, and 85 cases obtained the clinical outcome, with the last update in June 2011. The median follow up time was 80 months.

### RT-PCR

Total RNA from cells was extracted using TRIzol regent (Gibco Invitrogen, Carlsbad, CA, USA) following the manufacturer’s instructions, converted to cDNA with RevertAid First Strand cDNA Synthesis Kit (MBI Fermentas, Vilnius, Lithuania). Classical RT-PCR amplification was undertaken with Taq DNA Polymerase (MBI Fermentas, Vilnius, Lithuania). The primer sequences for DKK1 were: forward AACGCTATCAAGAACCTGC and reverse GATGACCGGAGACAAACA resulting in 460-bp amplification. The primer sequences for GAPDH were : forward GGGAGCCAAAAGGGTCATCATCTC and reverse CCATGCCAGTGAGCTTCCCGTTC, resulting in a 353 bp amplification. The cycling conditions were 5 min at 94°C, 24 (GAPDH) or 35 (DKK1) cycles of 30 s at 94°C, 45 s at 52°C, 1 min at 72°C, with a final extension of 10 min at 72°C, in the Eppendorf Mastercycler 22331 (Eppendorf, Hamburg, Germany).

### Western Blot

Cell lysates were clarified by centrifuge at 10,000 g. Immunoblots were probed according to standard protocols with antibody DKK-1 (Santa Cruz Biotechnology, Santa Cruz, CA, USA) and beta-catenin (Abcam, Cambridge, MA, USA). The quality of loading and transfer was assessed by immunostaning with GAPDH antibody (Santa Cruz Biotechnology, Santa Cruz, CA, USA). Immunoblots were developed by using enhanced chemilimminescent regent (Pierce ECL, Rockford, IL, USA) and images were captured by the FUJIFILM LAS-3000 system (Fuji film, Tokyo, Japan).

**Figure 2:**
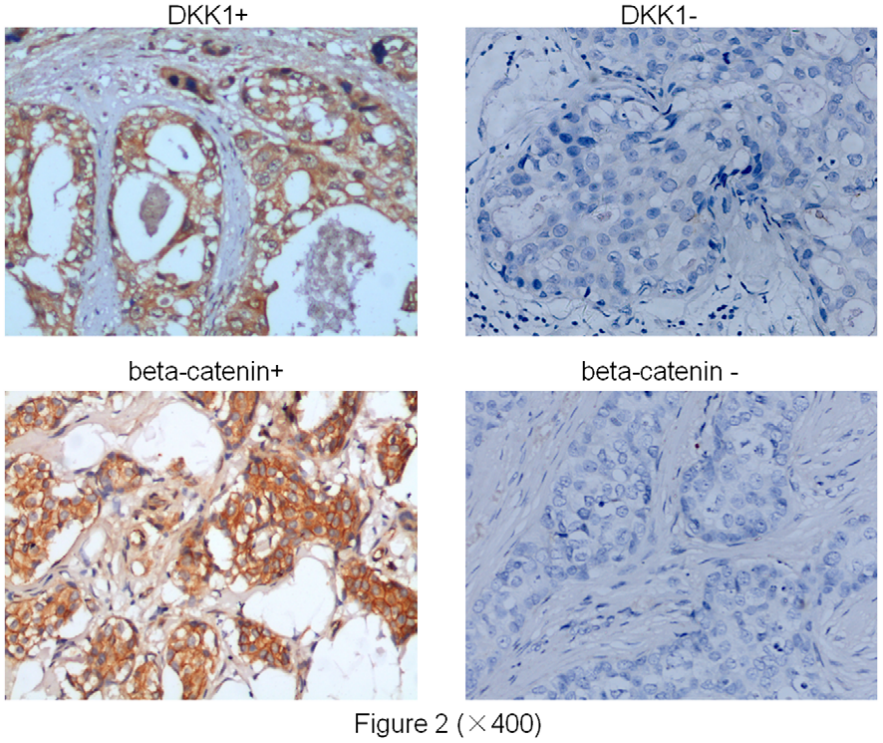
Cytoplasmic immunostaining of DKK1 and beta-catenin in carcinomas. A) Positive immunostaining of DKK1 in an invasive ductal breast carcinoma, ×400 magnification. B)Negative immunostaining of DKK1 in another invasive ductal breast carcinoma, ×400 magnification. C) Positive immunostaining of beta-catenin in an invasive ductal breast carcinoma, ×400 magnification. D) Negative immunostaining of beta-catenin in another invasive ductal breast carcinoma, ×400 magnification.

**Table 2:** Correlation between the expression of DKK1 and beta-catenin.

	DKK1(+)	DKK1(-)	Total
Beta-catenin(+)	44(51.76%)	11(12.94%)	55
Beta-catenin(-)	8(9.41%)	22(25.88%)	30
Total	52	33	85

X^2^ = 23.25, P = 0.000.

### Immunohistochemistry

Tumor sections were subjected to immunohistochemical staining for DKK1 and beta-catenin. Antibodies used were polyclonal DKK1 antibody (Santa Cruz Biotechnology, Santa Cruz, CA, USA) and polyclonal beta-catenin antibody (Abcam, Cambridge, MA, USA ). Tumor sections were incubated in a 1∶50 dilution of DKKA and a 1∶1000 of beta-catenin at 4°C overnight. Primary antibodies were detected by HRP-conjugated secondary antibodies followed by colorimetric detection with 3,3-diaminobenzidine (DAB).

**Figure 3:**
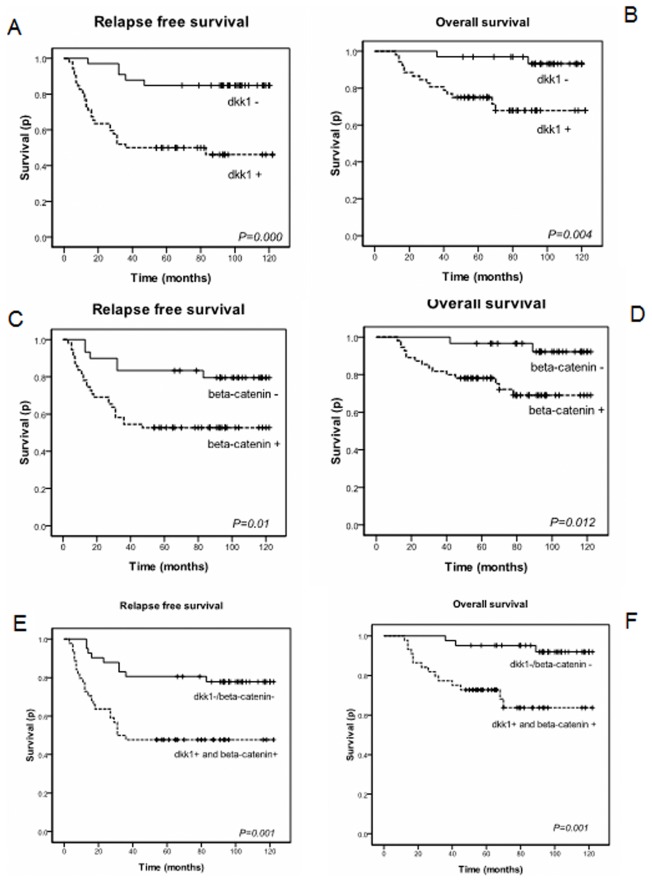
Kaplan-Meier curves based on DKK1 and beta-catenin expression. A) B): Kaplan-Meier estimates for relapse-free survival and overall survival based on DKK1 status. C) D): Kaplan-Meier estimates for relapse-free survival and overall survival based on beta-catenin status. E) F): Kaplan-Meier estimates for relapse-free survival and overall survival based on DKK1 and beta-catenin status.

**Table 3:** Univariate analysis of prognostic factors for breast cancer patients.

	Relapse-free Survival		Overall Survival	
	RR(95%CI)	P	RR(95%CI)	P
Pathology type	0.794(0.367–1.716)	0.557	0.624(0.231–1.688)	0.353
Lymph node status	2.294(1.139–4.621)	0.020	1.943(0.748–5.049)	0.173
Tumor size	1.651(0.763–3.570)	0.215	1.444(0.509–4.102)	0.490
Tumor stage	3.510(1.743–7.066)	0.000	3.369(1.289–8.807)	0.013
Menopausal status	1.772(0.866–3.626)	0.117	1.726(0.638–4.667)	0.282
dkk1	4.898(1.878–12.772)	0.001	7.104(1.595–31.642)	0.010
beta-catenin	3.035(1.246–7.389)	0.014	5.482(1.243–24.182)	0.025

**Table 4:** Multivariate analysis of prognostic factors and adjuvant treatments for breast cancer patients.

	Relapse-free Survival		Overall Survival	
	RR(95%CI)	P	RR(95%CI)	P
Lymphnode status	1.105(0.433–2.820)	0.835	-	-
Tumorstage	2.539(0.993–6.487)	0.052	2.773(1.032–7.449)	0.043
dkk1	3.048(1.060–8.765)	0.039	3.756(0.780–18.077)	0.099
beta-catenin	1.751(0.664–4.617)	0.257	3.392(0.708–16.245)	0.126

**Table 5:** Prognostic value of DKK1 in stage II tumor, lymph node negative subgroup.

		OS		P(logrank test)	dfs		P(logrank test)
		0	1		0	1	
Stage II	DKK1+	20	2	0.028	19	3	0.013
	DKK1−	21	7		16	12	
Lymph node negative	DKK1+	22	2	0.023	22	2	0.002
	DKK1−	20	6		14	12	

0: if event censored; 1: if event occurred.

### Evaluation of Immunohistochemical Variables

Immunohistochemical staining results were assessed by two pathologists independently with no knowledge of patient characteristics. Discrepancies were resolved by consensus. DKK1 staining was evaluated using the following criteria: positive, dark brown staining in >50% tumor cells completely obscuring cytoplasm; and absent, no appreciable staining in tumor cells [21]. For beta-catenin protein immunoreaction evaluation, we considered more than 10% of cells exhibiting cytoplasmic/nuclear staining as positive results [22].

**Table 6:** Correlation of DKK1 and beta-catenin expression with other clinical and pathological parameters.

	DKK1+	DKK1-	X^2^	P	beta- catenin+	beta- catenin-	X^2^	P
Pathology type								
IDC	37	26	1.204	0.273	45	18	4.817	0.028
Other	15	7			10	12		
Lymph node status								
0	26	24	4.305	0.038	31	19	0.389	0.533
> = 1	26	9			24	11		
Tumor size								
< = 2 cm	22	11	0.685	0.408	23	11	0.215	0.643
>2 cm	30	22			32	19		
Tumor stage								
Stage1,2 = 0	33	29	6.098	0.014	37	25	2.537	0.111
Stage 3 = 1	19	4			18	5		
Menopausal status								
Pre	25	16	0.001	0.971	24	17	1.320	0.251
Post	27	17			31	13		

### Statistical Analysis

The follow-up period was defined as the time from surgery to the last observation for censored cases or relapse/death for complete observations. Relapses-free survival was defined as the time between the date of the primary surgery to the date of relapse or June 2011. Overall survival was defined as the time between the date of the primary surgery to the date of death or June 2011. Postoperative relapse-free survival probability and overall survival probability were derived from the Kaplan-Meier estimate, and the differences between survival curves were compared by means of the logrank test. DKK-1 positive tumors were compared to DKK-1 negative tumors for the same clusters, using the two sided Person x^2^ test. Statistics was analyzed using Stata/SE version 10.0 (Stata Corporation, College Station, TX, USA). All P values are two-sided and P<0.05 were considered significant.

## Results

### 1. Expression of DKK1 in Breast Cell Lines

The levels of DKK1 mRNA in 6 different breast carcinoma cell lines were compared with those in the normal MCF10A breast cell line and in Hela cell line. The mRNA expression of DKK-1 in MCF-7, SK, MDA-MB-435 and Hela cell lines were relatively lower than those in BT474, MDA-MB231, MDA-MB231-HM and MCF10A cells. We further investigated DKK1 protein expression in these cells by western-blot. As shown in Figure 1, high DKK1 protein expression was confirmed in hormone-resistant breast cell lines MDA-MB231, MDA-MB231-HM, MDA-MB-435 expect for SK. Interestingly,a very low expression level of DKK1 was found in MCF-7 and BT474 cells, which were both hormone sensitive. And a low amount of DKK1 was also found in MCF10A cell.

### 2. Patient Characteristics

Next we investigated the relationship between DKK1 and beta-catenin expression level in clinical breast cancer samples by immunohistochemical staining. All of the tumor samples were invasive breast carcinomas. Fifty of the cases had undergone modified radical mastectomy, 29 radical mastectomy, and the remaining 6 cases breast-conserving surgery. After the surgical procedure, 72 patients received adjuvant systemic therapy according to accepted practice guidelines. Chemotherapy only was administered to 55 patients, 17 received radiotherapy after chemotherapy, and 10 of the patients were given hormonal therapy. The chemotherapy regimen for most of these patients was mainly anthracycline based. Patient characteristics are presented in Table 1. During the follow-up, 64 patients (75.29%) were disease free without relapse or metastasis, 32 (37.65%) patients developed recurrence or metastasis: 8 (9.41%) with local relapse and 24 (28.24%) with bone or distant metastasis (liver, lung, brain) and 17 (20.00%) patients of those died of breast cancer.

### 3. DKK1 and Beta-catenin Immunostaining

Representative images of DKK1 and beta-catenin were listed in Figure 2. DKK1 staining was mainly observed in the cytoplasm of tumor cells. Expression of DKK1 was found in 52 cases of all the 85 cases, negative expression was found in 33 cases. For the cellular localization of beta-catenin by immunostaining, cytoplasmic/nuclear staining of beta-catenin was observed in 55 of 85 breast cancer patients, and no staining was observed in the other 30 cases. And there was a significant correlation between the expression of DKK1 and beta-catenin (p = 0.000). Details were listed in Table 2.

### 4. Association of DKK1 and Beta-catenin with Clinical Outcome in Triple Negative Patients

Using the logrank test, Figure 3A-B showed significant differences in relapse-free survival curves and overall survival curves according to DKK1 expression. Patients with negative DKK1 expression had a better relapse free survival than those with positive expression (84.8% vs 48.1%, P = 0.000). Overall survival difference was also observed between the DKK1 negative and positive patients (71.2% vs 93.9%, P = 0.004). Figure 3C-D presented survival curves according to beta-catenin expression. The relapse-free survival probability of beta-catenin negative patients was better than beta-catenin positive patients (80% vs 52.7%, P = 0.01). Statistical difference in overall survival was seen between beta-catenin-negative and beta-catenin-positive patients (93.3% vs 72.7%, P = 0.012). Patients with either DKK1 negative or beta-catenin negative had better relapse free survival than patients with both DKK1 and beta-catenin positive expression (78% vs 47.7%, P = 0.001). Statistical difference was also observed in overall survival (92.7% vs 68.2%, P = 0.001).

### 5. Univariate and Multivariate Analysis of Prognostic Factors and Adjuvant Treatments for Breast Cancer Patients

Univariate analysis of relationships between tumor characteristics and patients’ outcome indicated that lymph node status, tumor stage, expression of DKK1 and beta-catenin were significantly associated with relapse-free survival, whereas no significant prognostic values were found with other factors listed in Table 3. As for overall survival, tumor stage, DKK1 and beta-catenin were found to have significant prognostic values while no such correlation was found in other factors (Table 3). Cox’s proportional-hazard regression model was used to determine which factor was independently or jointly predicative of relapse-free survival and overall survival. Multivariate analysis indicated DKK1 to be an independent risk factor for relapse free survival and tumor stage to be an independent risk factor for overall survival (Table 4). We further tested the prognostic value of DKK1 in different tumor stage or lymph node status subgroups. We found that in our study the DFS and OS of stage II DKK1 negative tumors was significantly higher than that of DKK1 positive tumors (DFS p = 0.013; OS p = 0.028). In the lymph node negative group, DFS and OS of DKK1 positive patients was significantly lower than that of DKK1 negative patients (DFS p = 0.002; OS p = 0.023). Details were listed in Table 5.

### 6. Correlation of DKK1 and Beta-catenin Expression with Other Clinical and Pathological Parameters

Table 6 listed the correlation of DKK1 and beta-catenin expression with the tumor characteristic parameters. The expression of DKK1 was found associated with the lymph node status, tumor stage and the expression of beta-catenin was associated with pathology type. No significant association was found in other tumor parameters.

## Discussion

In this study we reported that DKK1 protein was expressed in breast cancer hormone-resistant cell lines MDA-MB-231, MDA-MB-231-HM and MDA-MB-435. Although DKK1 mRNA expression was detected in receptor-positive or HER2-positive breast cancer cells we studied, no such protein expression was found in these cells. It is likely that most DKK1 has already been secreted out of these cells and so no DKK1 can be detected in the cells. Forget MA [19] has reported positive expression of DKK1 in the supernatants of MCF-7 cells by ELISA.

We found patients with positive expression of DKK1 as well as beta-catenin were associated with a poor outcome, meanwhile the DFS and OS of stage II DKK1 negative tumors in our study was significantly longer than that of DKK1 positive tumors(DFS p = 0.013; OS p = 0.028). Therefore, it might be helpful to integrate the DKK1 expression status with the tumor stage to help predict the prognosis of triple negative breast cancers, although the cases in other stages were too small to detect its prognostic significance. Furthermore, in patients with no lymph node metastasis, the DFS and OS of DKK1 positive patients was significantly longer than that of DKK1 negative patients(DFS p = 0.002; OS p = 0.023). Thus DKK1 expression might be helpful to predict the prognosis of node-negative patients.

DKK1, which interacts with LPR6 of Wnt/beta-catenin pathway, is regulated by progesterone in normal endometrical stroma cells [23]. Consistent with our result, Forget MA [19] and co-workers had also observed DKK1 in two hormone-independent prostate cancer lines (DU45 and PC3) and hormone receptor-negative breast tumor but not in a hormone-dependent tumor (LNCaP). Up-regulation of DKK1 in our study correlated with cytoplasmic/nuclear beta-catenin accumulation in triple negative breast cancers (p = 0.000). As DKK1 is a Wnt target gene, this elevation may actually indicate the activation of pathway activity.

The role of the Wnt/beta-catenin pathway activation in breast cancer has been controversial. Beta-catenin plays significant roles in the regulation of mammary development and tumorigenesis through its association with E-cadherin in cell adhesion, regulation of mammary stem cells and Wnt/beta-catenin signaling pathway. Lin SY and coworkers reported the nuclear staining of beta-catenin was associated with poor outcome of breast cancer patients [11]. However, several other studies failed to find such association [24], [25]. One possible explanation is these studies did not categorize the beta-catenin localization by molecular subtypes. Khramtsov AI and colleagues previously reported that Wnt/beta-catenin pathway activation was enriched in basal-like breast cancers [13]. There are significant limitations to this current study, including the small data set, few cases of stage I patients, no validation in an independent series of cases, categorize the accumulation of DKK1 and beta-catenin in breast cancers by immunohistochemical to positive and negative rather than scoring. Despite these shortcomings, our results do support the idea that Wnt/beta-catenin signaling pathway is activated in triple negative breast cancers.

Although triple negative breast cancers only account for 15–20% of all breast cancers, it represents an important clinical challenge since no endocrine therapy or targeted agents are available. Current treatment strategies for it include many chemotherapy agents. Comparing with luminal As or Bs, there is a markedly higher response rate to chemotherapy in this subtype. However, the disease-free survival and over-all survival of triple negative breast cancers are far shorter. Our data indicated that beta-catenin might be a potential therapeutic target of this subtype. Effects of Wnt pathway inhibitors have been tested in colorectal cancer [26], [27], further investigation of such compounds are necessary to be carried out *in vitro* and *in vivo* to explore their potential therapeutic effects in triple negative breast cancers.

In conclusion, accumulation of DKK1 and cytoplasmic/nuclear beta-catenin in triple negative cancers indicate poor outcome of patients. DKK1 might be a valuable biomarker in predicting the prognosis of patients with earlier stage or no lymph node metastasis. DKK1 alone or together with beta-catenin might be helpful in identifying patients who might benefit from early systemic treatment. The potential mechanism between increased DKK1 expression and Wnt/beta-catenin pathway activation and the mechanism by which the Wnt/beta-catenin pathway is activated in triple negative cancers is still unknown. It is possible that through further understanding of the role of Wnt/beta-catenin pathway activation, beta-catenin would be a potential therapeutic target for the triple negative breast cancer.
