# Correction: Expression of Dickkopf-1 and Beta-Catenin Related to the Prognosis of Breast Cancer Patients with Triple Negative Phenotype

**DOI:** 10.1371/journal.pone.0102404

**Published:** 2014-07-03

**Authors:** The PLOS ONE Staff

There is an error in the equal contributions designation. The first two authors, Wen-Huan Xu and Zhe-Bin Liu, should be noted as contributing equally to this work.
